# Curriculum Innovation: *ABCs of Child Neurology*: An Article-Based Curriculum to Strengthen Outpatient Training Through Self-Directed Learning

**DOI:** 10.1212/NE9.0000000000200327

**Published:** 2026-06-17

**Authors:** Elizabeth T. Troy, Jan Martin, Michelle Tutmaher, Hannah Gilbert, John Binder, Lori Silveira, Megan Abbott, Timothy J. Bernard, Scott Lance Rosenthal, Aaron M. Carlson, Jeremy J. Moeller, Tai Lockspeiser

**Affiliations:** 1 Section of Neurology, Department of Pediatrics, University of Colorado School of Medicine, Aurora;; 2 Division of Pediatric Neurology, Department of Neurology, University of Washington School of Medicine, Seattle;; 3 Division of Neurology, Department of Pediatrics, Emory University School of Medicine, Atlanta, GA;; 4 Department of Pediatrics - Biostatistics, University of Colorado School of Medicine, Aurora;; 5 Department of Neurology, University of Colorado School of Medicine, Aurora;; 6 Department of Neurology, Yale School of Medicine, New Haven, CT; and; 7 Department of Pediatrics, University of Colorado School of Medicine, Aurora.

## Abstract

**Introduction and Problem Statement:**

Establishing a foundation of outpatient knowledge is key for child neurology residents as they prepare for careers shaped by evolving diagnostics and treatments. Although national recommendations define core didactic content, strategies for implementation vary widely. To address this gap, educators developed the *ABCs of Child Neurology (ABCs)*, an asynchronous, article-based curriculum grounded in the Master Adaptive Learner framework.

**Objectives:**

On participation, learners will (1) develop competency in outpatient child neurology foundational principles and (2) cultivate self-directed learning behaviors through an asynchronous curriculum.

**Methods and Curriculum Description:**

*ABCs* is mirrored after the American Board of Psychiatry and Neurology (ABPN) Article-Based Continuing Certification program. It comprises 40 modules across 8 core outpatient topics, each featuring a high-yield clinical article and 5-item assessment. The curriculum was implemented during the 2024–2025 academic year for child neurology residents (PGY3–PGY5) at a single institution. Learner expectations include completing 1 module per month and scoring ≥80% on the assessment to receive credit. Evaluation follows the New World Kirkpatrick model, evaluating learner reaction and knowledge growth. Outcomes include module completion rates, aggregate performance on module assessments, and postmodule and postintervention surveys. Descriptive statistics and subgroup analyses by postgraduate year were performed.

**Results and Assessment Data:**

Ten residents (100%) participated in the pilot. Overall completion rate was 97%, with individual rates ranging from 75% to 107%. Of 129 completed modules, 116 (89.9%) were passed on the first attempt. Mean assessment score was 4.4/5 (SD = 0.8), and median completion time was 43 minutes (IQR-17-470 minutes). Patient care was the most common motivator (41.9%). Postmodule survey ratings revealed 95.7% (mean = 4.5/5; SD = 0.6) of content as essential to practice and 94% (mean = 4.5/5; SD = 0.6) reporting likelihood to apply material. Ratings increased across training levels—PGY-3 = 4.0 (SD = 1.1), PGY-4 = 4.4 (SD = 0.8), and PGY-5 = 4.7 (SD = 0.6) (*p* = 0.008). Postintervention surveys indicated strong endorsement of the curriculum to colleagues (4.5/5; SD = 0.7; 90% rating 4 or 5).

**Discussion and Lessons Learned:**

Early work at a single institution supports the feasibility and quality of an asynchronous, article-based curriculum for outpatient child neurology education. Key lessons from the pilot included prioritizing usability to support sustained engagement and aligning content with clinical responsibilities. Future work will evaluate scalability across multiple institutions and explore qualitative insights to optimize integration of asynchronous curricula.

## Introduction and Problem Statement

Outpatient clinical care is a cornerstone of child neurology practice and, thus, a fundamental component of the Accreditation Council for Graduate Medical Education Child Neurology Milestones^1^ and American Board of Psychiatry and Neurology (ABPN) Core Competencies.^2^ Collectively, these standards underscore that ambulatory learning is essential within child neurology training. National data demonstrate a persistent asymmetry between inpatient and outpatient clinical experience within both adult and child neurology residency programs, highlighting a mismatch between training exposure and practice needs.^3^ At the University of Colorado Child Neurology Residency Program, an internal review identified that this mismatch extended to our didactic curriculum as well. Furthermore, the prior article-based learning materials consisted primarily of printed papers stored in a drawer or intermittently collected in a shared electronic folder—resources that were accumulated over time but rarely curated or updated, resulting in inconsistent access, uneven use, and no mechanism to assess competency with the content.

Coverage of outpatient neurology content varies widely across programs, as does the approach to teaching it. Traditional lecture-based didactics have long dominated graduate medical education (GME), yet they often fail to engage adult learners or foster active knowledge application.^4^ A scoping review of neurology education literature shows that e-learning has grown steadily in popularity and, in the final year of analysis, was tied with simulation as the most common educational innovation.^5^ Notably, this same review found fewer than 10 of 350 publications focused on child neurology, underscoring a significant gap in structured education within the discipline. Despite the growing emphasis on technology-enhanced learning,^6^ resources tailored to child neurology remain scarce with most tools designed for adult neurology trainees. Existing guidelines from the American Academy of Neurology address subspecialties such as neuropalliative care,^7^ psychiatry,^8^ sleep,^9^ and neuroradiology^10^; however, pediatric objectives are minimal or absent. Similarly, widely used neurology textbooks often only include a single section on child neurology.^11,12^ Collectively, these findings highlight the urgent need for a standardized, evidence-based outpatient curriculum in child neurology.

Self-directed learning (SDL) is central to contemporary health professions education, requiring learners to “take initiative for their own learning.”^13^ This developmental framework provides a structure for building essential outpatient knowledge while also cultivating habits that can translate to lifelong autonomous practice. The ABPN's Article-Based Continuing Certification (ABCC) program^14^ exemplifies this principle by requiring diplomats to engage in continuous professional development through structured review of sentinel articles and completion of competency-based assessments. This model ensures physicians remain current with evolving literature, promoting evidence-based practice throughout their careers. Embedding similar strategies within residency education offers an opportunity to cultivate these habits early, equipping trainees to adapt to evolving clinical knowledge throughout their careers.

Building on these principles, the *ABCs of Child Neurology* was developed as a self-directed, article-based curriculum designed to provide a foundational framework for outpatient child neurology education. The curriculum applies SDL principles through the Master Adaptive Learner (MAL) framework, which is a conceptual framework that promotes adaptive expertise by guiding learners through 4 iterative phases of planning, learning, assessing, and adjusting.^15^ In this model, residents engage in a structured cycle of self-reflection on knowledge gaps, focused learning using curated articles, and assessment and integration of new knowledge into clinical practice. By embedding MAL into an article-based platform, the *ABCs* curriculum moves beyond traditional didactics to align with competency-based educational standards and modern learning theory. The aim of this study is to describe the development of this innovative curriculum and evaluate its feasibility and quality during pilot implementation at a single institution.

## Objectives

After completion of the curriculum, learners will:

1. Develop foundational competency in ABPN Child Neurology Core Competencies by engaging with sentinel articles and demonstrating applied understanding on structured assessments
2. Cultivate self-directed learning behaviors by independently selecting relevant literature and synthesizing the material to support clinical decision making in the ambulatory setting.

## Methods and Curriculum Description

### Stakeholder Engagement

In preparation for a grant application, the lead author emailed child neurology residency program directors using publicly available contact information from the Child Neurology Society website. In this outreach, program directors were invited to sign a letter of support acknowledging a gap in existing outpatient educational methods within child neurology and expressing the potential value of a national, article-based, asynchronous curriculum to address this need.

### Curriculum Design

**Figure 1:**
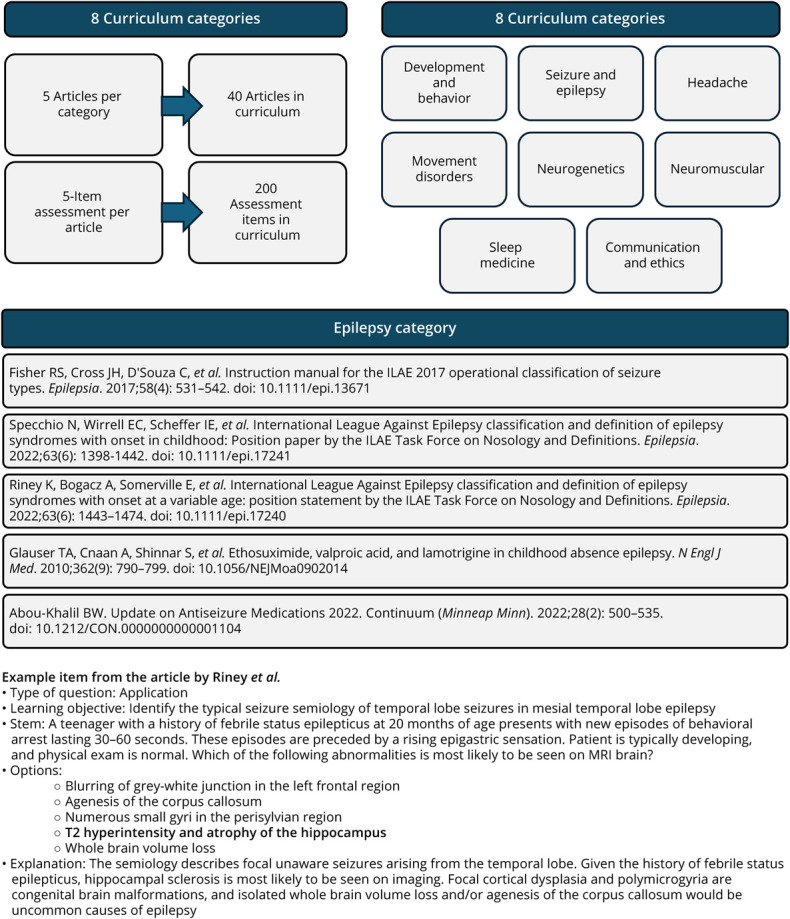
Overall Curriculum Structure: Curriculum Categories, Articles in the Epilepsy Category, and Example Structure of Items

*ABCs of Child Neurology* is modeled after the ABPN ABCC program. It is comprised of 40 modules across 8 core outpatient topics, and each module features a high-yield clinical article and a corresponding 5-item knowledge assessment (Figure 1). A team of 13 experienced educators, representing 4 academic institutions and fields of pediatrics, child neurology, and adult neurology, was assembled to develop *ABCs*. The committee reviewed the ABPN Child Neurology Core Competencies^2^ and reached consensus on the final 8 topics most aligned with an outpatient curriculum (Figure 1). Committee members conducted literature reviews to identify sentinel articles for each topic, prioritizing publications from the past 5 years and those featuring practice parameters or symptom-based management algorithms. For each topic, 7–8 articles were initially selected, and the final 5 were chosen through group consensus. Figure 1 presents the 5 articles selected for the Epilepsy category. A complete Article Dictionary containing all article citations and their associated learning objectives is provided in the Supplementary Material (eAppendix 1).

Each article was paired with a clinically relevant 5-item multiple-choice assessment. Writers received training in National Board of Medical Examiners (NBME) best practices for item writing.^16^ Most items were structured as clinical vignettes with 4–5 answer choices, followed by an explanation of the best answer. Each item was developed to assess a specific, predefined learning objective (eAppendix 1). Figure 1 includes an example item to illustrate assessment style. Items underwent review in read-aloud groups and were further refined by an item-writing expert before publication.

The finalized curriculum was implemented using the Qualtrics platform (Provo, UT). To encourage self-reflection, participants indicated their motivation for selecting each topic and specific article and identified a key learning point. Initially, participants received unique links for each module; however, because of operational challenges, this was later streamlined to a single open-access link for the residency program with a name field to track participation. Throughout the year, residents accessed articles in 2 ways. In the pilot, articles were available as integrated PDFs within Qualtrics, and in the second half of the year, they were also provided in a shared electronic folder to improve accessibility.

### Pilot Implementation

The pilot curriculum was launched during the 2024–2025 academic year in the University of Colorado Child Neurology Residency Program. All postgraduate year (PGY) 3–5 child neurology residents were eligible to participate. The curriculum itself was considered an important element of our educational curriculum, and thus, all residents were expected to complete the expected modules. Participation in the research elements, specifically the pre- and postassessments and surveys as described below, was voluntary. The curriculum was introduced at the annual residency retreat in May 2024 and reiterated during the first didactic session in July 2024. Residents were expected to complete 1 module per month and achieve a minimum assessment score of 80% to receive credit. Given the variability in clinical responsibilities, progress was tracked quarterly. Participants were required to complete at least 1 module from each of the 8 topic areas over the academic year. Reminder emails were sent 2 weeks and 48 hours before the end of each quarter.

Over the academic year, self-directed learning time was gradually integrated into the curriculum to support development of this core competency. Residents were encouraged to schedule a half day of self-directed learning during outpatient and elective blocks. In addition, child neurology residents attended the weekly adult neurology academic half day, and because not all sessions were equally applicable to their training needs, residents were permitted to use selected sessions for targeted self-directed learning to address individual knowledge gaps. In parallel, residents continued to participate in the program's established educational activities, including weekly Tuesday case conferences, weekly Friday faculty-led child neurology didactics, and the monthly child neurology academic half day.

### Program Evaluation

Curriculum participants completed identical pre- and post-knowledge assessments at the start and end of the academic year. The assessment consisted of 40 items, with 1 representative question selected from each module. The pre-assessment was completed before residents had access to the curriculum. At the end of the academic year, residents completed the postassessment. Because the postassessment reused the item associated with any module a resident completed, the number of previously seen items varied. For example, a resident who completed 12 modules would have encountered the 12 familiar items on the postassessment, while the remaining 28 items were novel. Two surveys were developed to evaluate the curriculum—one to be completed after each module and a second completed at the end of the year. Survey item development was guided by Artino's *AM Last Page*.^17^ At the end of each module, participants completed a 4-item survey evaluating satisfaction and perceived quality. A separate postintervention survey was designed to evaluate overall curriculum quality and impact. Both surveys underwent expert validation by committee review. The postintervention survey was pilot tested by 2 participating senior child neurology residents who confirmed the clarity, intent, and alignment of answer options.

Evaluation was guided by the New World Kirkpatrick model^18^ using a structured quantitative approach. Level 1 (Reaction and Engagement) outcomes were evaluated through postmodule and postcurriculum survey responses, as well as measures of resident engagement including quarterly module completion, motivation for engagement, and frequency of topic and article selection. Level 2 (Learning) outcomes included aggregate knowledge assessment scores and pre- to postassessment comparisons. Together, these complementary measures provided triangulated evidence of curriculum impact across multiple levels of the Kirkpatrick framework.

To safeguard resident anonymity and reduce the risk of perceived pressure, all participant data were deidentified before review by the research team. Although residents entered their names into the learning platform when completing the modules and knowledge assessments, an education coordinator removed all identifiers before data analysis. As a result, investigators had no access to individual-level identifiable responses, maintaining the integrity of the program evaluation process.

### Statistical Analysis

Descriptive statistics were summarized as proportions, percentages, or medians with standard deviation or interquartile range where appropriate. Module completion was calculated as the number of modules completed divided by the expected 3 modules per quarter or 12 modules per year. Completion percentages could exceed 100% when residents completed more than the expected number of modules. Time to complete each module was measured in Qualtrics as the interval between opening the module link and submitting the assessment and postmodule survey. Percent-correct scores were calculated for each assessment. In addition, the raw pre- and postassessment scores and computed percentage point differences were analyzed between pre- and postperformance for each resident. These values were derived for both the full assessment and the subset of assessment items corresponding to modules the resident completed during the curriculum. For the postmodule survey analyses, only first-attempt responses were included. Each module's survey contained 4 Likert-scale questions. These were first averaged within each module, then aggregated within by each category, and then finally summarized for the curriculum as a whole.

To examine subgroup effects, data were stratified by postgraduate year, and differences between years were compared. For the postmodule survey, each resident's averaged first-attempt survey scores were aggregated across completed modules, and differences among PGY were compared using Fisher exact test. For the assessment data, pre- and postassessment scores were paired, and comparisons were conducted using the Wilcoxon signed rank test for comparing differences as a whole and then by Kruskal-Wallis tests when analyzing as groups.

All statistical tests were 2-tailed with significance set at *p* < 0.05. Analyses were conducted using SAS 9.4 (SAS Institute Inc., Cary, NC).

### Standard Protocol Approvals, Registrations, and Participant Consents

This study was approved by the University of Colorado Institutional Review Board under Protocol 24–0920 as an exempt study. Eligible residents received an IRB-approved information sheet within the Qualtrics email invitation, and informed consent was documented when the residents reviewed the study information and clicked the secure link, granting access to the curriculum.

### Data Availability

Curriculum materials and deidentified data will be made available upon request by a qualified educator.

### Artificial Intelligence Disclosure Statement

This article represents the original work of the authors. Aside from minor item editing, artificial intelligence tools were not used in the design of the curriculum, and all described content was developed independently by the authors. Microsoft Copilot was used solely to enhance the clarity and flow of the written text. The authors have reviewed the final article and affirm its readiness for publication.

## Results and Assessment Data

### Stakeholder Response

Of the roughly 70 program directors contacted during stakeholder outreach, 17 signed the letter indicating support for a structured outpatient curriculum, representing approximately one quarter of child neurology programs nationally. These directors also expressed interest in participating as the curriculum expands.

### Descriptive Analysis

Eleven residents were enrolled in the pilot curriculum, and all residents chose to participate in the research elements. One resident was excluded from the analysis because of an extended leave, resulting in a final cohort of 10 residents, specifically 2 PGY-3, 4 PGY-4, and 4 PGY-5 residents.

Across the academic year, residents completed 129 modules, of which 116 (89.9%) were successfully passed with an assessment score ≥80%. When calculated at the program level, the residency achieved an overall completion rate of 96.6% (116 of 120 expected modules). Individual resident completion rates ranged from 75% (9 of 12 expected modules) to 108% (13 of 12 expected completed modules). The median time to complete a module in the first attempt was 43 minutes with an interquartile range of 17–470 minutes. Table 1 provides additional details by quarterly data. Regarding motivation for category selection, the most frequently cited reason was an upcoming or recent patient interaction (49/117; 41.9%), followed by no clear reason (32/117; 27.4%), already covered other categories (24/117; 20.5%), and other (12/117; 10.3%). Among the 12 free-text “other” responses, most reflected a perceived personal knowledge gap or a broader curriculum gap in formal training while a small number cited a recent or upcoming clinical or didactic exposures as the reason for a category selection.

**Table 1:** Curriculum Utilization in the Pilot Curriculum

10 Residents	Q1	Q2	Q3	Q4	Total
Total Modules	35	32	31	31	129
Passing Modules	32	29	28	27	116
Completion Rate	107%	97%	93%	90%	97%
Score in the First Attempt (Mean)	90%	89%	90%	87%	89%
Time to Complete the First Attempt (Median)	90 min	57 min	25 min	20 min	43 min

All 8 categories were used during the pilot. Neuromuscular disorders accounted for the highest proportion of completed modules (22/117; 18.8%), while neurogenetics was the least used (10/117; 8.6%). Six of 10 residents (60%) completed at least 1 module across all 8 categories. Of the 40 articles, 38 (38/40; 95%) were completed by at least 1 participant. The mean score in the first assessment attempt was 4.4/5 with a standard deviation of 0.8, and as presented in Table 1, the mean score performance remained consistent across quarters.

**Figure 2:**
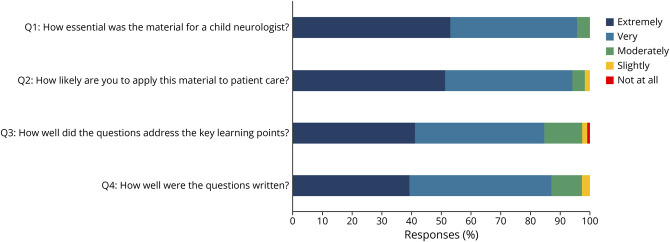
PostModule Survey of the Pilot Curriculum

Figure 2 displays the postmodule survey questions and corresponding results across all modules. Analysis included 117 first-attempt modules. Mean scores and Top Box percentages (responses of 4 or 5) for each question were as follows: essential material 4.5/5 (SD = 0.6, 95.7%), likelihood to apply material 4.5/5 (SD = 0.6, 94%), learning points 4.3/5 (SD = 0.7, 84.6%), and quality of questions written 4.3/5 (SD = 0.7, 87.2%). Table 2 provides a detailed breakdown of survey results by category, specifying postmodule survey results as well as further details on category-specific utilization and assessment scores.

**Table 2:** Category Utilization in the Pilot Curriculum

Category	Modules Completed	Mean Assessment Score (SD)	Essential Material Mean (SD, % Top Box^a^)	Apply to Patient Care Mean (SD, % Top Box^b^^a^)	Learning Objectives Mean (SD, % Top Box^b,^^a^)	Quality of Questions Mean (SD, % Top Box^b,^^a^)
Dev and Behavior^b^	12 (10.3%)	4.1 (0.9)	4.5 (0.7, 91.7%)	4.5 (0.7, 91.7%)	4.2 (0.8, 75%)	4.3 (0.7, 91.7%)
Comm and Ethics^c^	15 (12.9%)	4.7 (0.7)	4.5 (0.5, 100%)	4.5 (0.5, 100%)	4.5 (0.5, 100%)	4.5 (0.5, 100%)
Epilepsy	18 (15.5%)	4.6 (0.9)	4.7 (0.5, 100%)	4.5 (0.6, 94.4%)	4.3 (0.8, 77.8%)	4.3 (0.8, 83.3%)
Headache	11 (9.5%)	4.8 (0.4)	4.5 (0.5, 100%)	4.5 (0.5, 100%)	4.5 (0.5, 100%)	4.5 (0.5, 100%)
Movement	14 (12.1%)	4.5 (1.3)	4.5 (0.5, 92.9%)	4.4 (0.7, 85.7%)	4.2 (0.7, 92.9%)	3.7 (0.7, 85.7%)
Neuromuscular	22 (19%)	4.5 (0.7)	4.5 (0.7, 90.9%)	4.5 (0.6, 95.4%)	4.2 (0.7, 86.4%)	4.1 (0.6, 81.8%)
Neurogenetics	10 (8.6%)	3.9 (1.0)	4.5 (0.5, 100%)	4.4 (0.7, 100%)	4.1 (0.9, 70%)	4.3 (0.7, 80%)
Sleep	15 (12.9%)	4.3 (0.6)	4.3 (0.6, 93.3%)	4.3 (0.6, 86.7%)	3.9 (0.7, 73%)	4.1 (0.7, 80%)

Scale is 1 = not at all, 2 = slightly, 3 = moderately, 4 = very, 5 = extremely.

a Top Box is percentage selecting either 4 or 5.

b Child Development and Behavior.

c Communication and Ethics.

**Figure 3:**
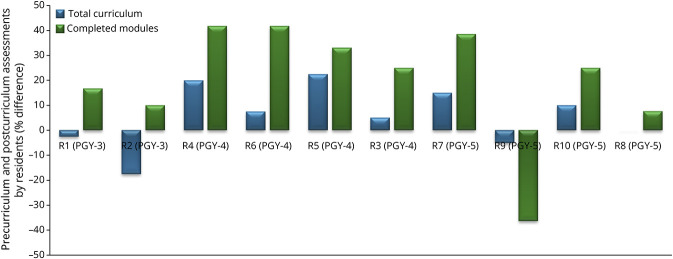
Percent Difference in Pre- and Postassessments, Stratified by Total Curriculum vs Completed Modules

The mean precurriculum assessment score was 50.3% (IQR: 45%–57.5%) compared with a postassessment mean of 57.5% (IQR: 50%–65%) (*p* = 0.172). Seven residents demonstrated equal or improved performance, while 3 showed a decline. When analysis was limited to questions from modules completed during the curriculum, residents answered an average of +2.4 additional questions (+20.7%) correctly on the postassessment. To further demonstrate individual-level variability, Figure 3 presents the percentage-point differences for each resident on both the full assessment (*p* = 0.172) and the subset of completed-module items (*p* = 0.033).

The postintervention survey assessed 3 domains: overall impression, curriculum structure, and operations. All residents (10/10, 100%) completed the survey. Table 3 presents mean scores and Top Box percentages for each item. The survey also included 2 open-ended questions, to which 5 residents provided a total of 9 comments. The first question asked residents to describe how the curriculum shaped their learning, and the second asked what modifications could better facilitate their learning. Representative narratives are included below to illustrate themes, and all qualitative responses are presented in full in the Supplementary Material

**Table 3:** Postintervention Survey

Category	Survey question	N = 10 Mean (SD, % Top Box^b^)
Overall	Which of the following best describes the quality of *ABCs*?^a^	4.2 (0.6, 90%)
	How effective was *ABCs* in improving your outpatient neurology knowledge?	3.8 (0.6, 70%)
	How likely are you to recommend this curriculum to a colleague?	4.5 (0.7, 90%)
Curriculum structure	How valuable was it to have a structured asynchronous curriculum as a component of residency training?	4.3 (0.7, 90%)
	How useful was it to have faculty-selected articles as a reference for learning?	4.7 (0.7, 90%)
	How feasible was the expectation of 12 articles per year?	4.4 (0.7, 90%)
Operations	How appropriate was the time required to complete 1 module?	4.2 (0.7, 90%)
	How easy was the platform to navigate?	4.3 (0.9, 90%)
	How easy was it to track your progress throughout the year?	3.5 (1.1, 60%)

Scale is 1 = not at All, 2 = slightly, 3 = moderately, 4 = very, and 5 = extremely.

a Unique scale for this question of 1 = unacceptable, 2 = borderline, 3 = acceptable, 4 = very good, and 5 = excellent.

b Top Box is percentage selecting either 4 or 5.

(eAppendix 2).

**Question 1**

The independent learning aspect was fantastic for my learning style to be able to do it at my own pace and on my own time.

I do not typically learn well by reading, being able to pair it with a recent or upcoming patient encounter worked best for me, which is how I know I learn best.

**Question 2**

…The questions after each article were occasionally too specific…it’s best to focus on the bigger picture points.

Having more faculty who work in education clinic be aware of articles and knowledgeable about them such that practice-based learning can happen more often.

### Comparative Analyses

Evaluation by training year revealed no significant differences in engagement across curriculum categories. When the pre-assessment and postassessment scores were stratified by postgraduate year, PGY-4 residents demonstrated the greatest improvement (+13.7%, SD = 8.8), while PGY-3 residents answered 4 fewer questions correctly (−10%, SD = 10.5) and PGY-5 residents answered 2 additional questions correctly (+5%, SD = 9.3) (*p* = 0.057). When this was further stratified by those modules completed in the curriculum, the median percent difference by year was PGY3 = −10% (IQR −17.5%, −2.5%), PGY4 = +15% (IQR 7.5%, 20%), and PGY5 = 0% (IQR -5%, 10%). Pairwise comparisons identified a significance between PGY-4 and PGY-5 (*p* = 0.049). First-attempt postmodule survey ratings, calculated as each resident's average of the 4 Likert-scale postmodule questions across all completed modules, also varied significantly by training level: PGY-3 = 4.0 (SD = 1.1), PGY-4 = 4.4 (SD = 0.8), and PGY-5 = 4.7 (SD = 0.6) (*p* = 0.008). Pairwise comparisons indicated that the difference between PGY-3 and PGY-5 was most pronounced (*p* = 0.002).

## Discussion and Lessons Learned

This study introduces *ABCs of Child Neurology* as the first comprehensive outpatient education platform for child neurology residents. Our findings demonstrate that a self-directed, asynchronous, article-based child neurology curriculum is feasible in GME. High module completion rates, consistent performance on module knowledge assessments, and strong learner endorsement underscore its acceptability and perceived value.

The *ABCs* curriculum operationalizes self-directed learning through the MAL framework by explicitly supporting each phase of the process—planning, learning, assessing, and adjusting. In the planning phase, patient care was the most frequently cited motivator for engagement, aligning with evidence that clinical encounters drive learning in GME.^19,20^ *ABCs* also addresses well-documented barriers in the planning stage, such as difficulty identifying credible sources and time constraints, by curating trusted, clinically relevant articles.^19^ During the learning and assessing phases, residents synthesize evidence and completed targeted questions, and the adjusting phase is supported by immediate feedback and reflected in high self-reported application of learning to patient care. Although MAL has been applied to procedural training^21^ and coaching,^22^ *ABCs* is, to our knowledge, the first application of MAL to an asynchronous curriculum in GME, offering a model that could inform curriculum development across other specialties.

By promoting self-directed learning within a structured framework, the *ABCs* curriculum not only strengthens outpatient clinical competency but also equips future child neurologists with the skills necessary for lifelong learning. This approach aligns with the American Board of Medical Specialties and ABPN Child Neurology Core Competencies,^2^ which emphasize identifying knowledge gaps through self-assessment and engaging in continuous learning. While these skills are difficult to measure directly, board certification and maintenance of certification (MOC) performance serve as surrogate indicators. MOC programs increasingly use longitudinal assessments and article-based programs, yet their application within GME remains limited. For example, spaced quizzes in a pediatric residency setting have demonstrated a positive correlation between biweekly quiz performance and certifying examination scores.^23^ By contrast, article-based strategies have only been implemented for practicing physicians.^24^ The ABPN piloted the ABCC program in 2019, and favorable feedback along with demonstrated clinical relevance^25^ led to its approval for all ABPN diplomats in 2022. *ABCs* introduces this model for the first time in residency training, preparing graduates to engage in lifelong learning and succeed as ABPN diplomats, a skill critical for sustained professional practice.

Resident feedback further highlights the curriculum's value while revealing opportunities for refinement. PGY-5 child neurology residents rated the curriculum most highly, likely reflecting their ability to contextualize its relevance to clinical practice and recognizing its role in addressing a significant curricular gap. PGY-4 residents demonstrated the greatest improvement in knowledge scores, consistent with their transition into child neurology as a primary focus during training. PGY-3 residents, whose responsibilities at our institution are largely concentrated on adult neurology, showed less benefit, suggesting the importance of congruence between clinical work and didactic curriculum. For the assessments, performance on module-level assessments was consistently high, whereas gains on the precurriculum to postcurriculum assessments were modest. Residents rated the curriculum highly overall in both the postmodule and postcurriculum surveys but assigned a comparatively lower score to its effectiveness. Open-ended responses on the postintervention survey indicated that some items were overly specific to nuances within the articles, which likely contributed to these observed assessment patterns. Future iterations should consider tiered content delivery, offering foundational modules for junior residents and advanced topics for senior trainees, and should also strengthen item quality to ensure that assessments accurately reflect the foundational learning the curriculum intends to promote.

Implementation evolved in response to resident input, leading to operational changes that influenced workflow and data trends. Early in the pilot, residents required individual access codes for each module, creating inefficiencies and perceived stigma when requesting replacements after failed attempts. Transitioning to an open-access curriculum link improved usability. Furthermore, articles were initially only accessible as PDFs within the platform, but residents requested centralized access, prompting creation of a shared folder with all readings. While this change also improved usability, it introduced limitations in interpreting time to module completion because residents accessed articles either before or after opening a Qualtrics module. Additional variability arose as some residents completed modules in one sitting, whereas others opened a module and returned later to finish it. Despite these factors, the median time to completion showed a consistent decrease across quarters, suggesting improved feasibility as residents became more comfortable with the platform. Nevertheless, the current platform Qualtrics lacks key features such as user-specific dashboards and longitudinal performance tracking. These limitations contributed to variability in completion rates and were reflected in survey feedback, where residents reported difficulty monitoring progress. Development of a dedicated learning management system is underway to further improve usability, promote self-directed learning, and automate performance tracking—ultimately strengthening the curriculum's scalability and sustainability.

Finally, *ABCs* reflects a broader shift in educational paradigms. Modern educational strategies emphasize not just the transfer of knowledge but also its application in clinical practice. While AI-supported tools are becoming increasingly integrated into medical education, their credibility varies widely. For this reason, it is essential for trainees to first build a strong foundation in vetted, peer-reviewed literature before relying on AI as a primary learning resource. The *ABCs* curriculum is designed to establish this foundational layer, enabling learners to later engage with AI tools critically and effectively, using them as adjuncts rather than substitutes for core knowledge. E-learning innovations, such as *ABCs*, are well suited for priming learners with core content that can then be reinforced through interactive sessions with expert faculty.^6^ This approach aligns with the visions outlined in *Neurology Education in 2035* where Moeller and Salas^26^ call for professional organizations to invest in a “shared curricular ecosystem” that enables institutions to distribute educational content. The *ABCs* curriculum represents an important step toward this vision and advances equity in neurology training.

## Limitations

This study has several limitations. As a pilot implemented at a single institution with a small sample size, the transferability of the curriculum remains uncertain. Institutional factors, such as scheduling flexibility and faculty engagement, may have influenced success. In addition, because the curriculum was led by the associate program director, residents may have felt encouraged to participate. While initial stakeholder engagement represented roughly one quarter of US programs, subsequent presentations of the curriculum have generated interest from 50 institutions, now representing well over half of all child neurology residency programs. Although there is national interest in adopting the curriculum, further research is needed to evaluate its integration into programs with diverse practice settings. Finally, this analysis focused on quantitative outcomes, limiting insight into the curriculum's impact on learning and clinical practice. Future work may be enhanced by incorporating qualitative data to capture learner perspectives and contextual factors influencing implementation.

## Next Steps

While initial feedback indicates that residents valued the *ABCs of Child Neurology*, future work must evaluate its broader educational and clinical impact. Focus groups conducted at the end of the academic year will serve as the primary method for assessing Kirkpatrick level 3 (Behavior) outcomes by exploring how the curriculum influenced patient care and supported self-directed learning among advanced trainees. These qualitative data will complement our quantitative findings and further elucidate how internal learner characteristics and the learning environment align with the MAL framework.

The curriculum is positioned for growth. During the current academic year, *ABCs* has expanded to 8 additional child neurology residency programs. This multicenter implementation will allow evaluation of scalability and adaptability across diverse educational environments, as well as identification of contextual factors that promote success. Concurrently, efforts are underway to expand existing categories with additional modules and introduce new topics to address the full breadth of child neurology. The long-term goal is to launch *ABCs* as an open-access national curriculum in July 2026, informed by lessons learned from this pilot and multicenter work.

Sustainability of the curriculum will be supported through a flexible but structured review process. An editorial board has been established in which 2 child neurologists oversee each topic area, enabling ongoing stewardship by faculty with domain-specific expertise. While the curriculum will remain nimble to allow updates throughout the year as new high-quality evidence emerges, a formal annual review will ensure systematic evaluation of all content, prioritizing review of articles older than 5 years, those infrequently selected by learners, and items demonstrating suboptimal psychometric performance. Together, with the planned development of a dedicated learning management system, this governance model positions *ABCs* to remain current, responsive, and scalable as it evolves into a national educational resource and advances a consistent, equitable framework for child neurology training.

## Glossary

ABPN: American Board of Psychiatry and Neurology
GME: graduate medical education
MAL: master adaptive learner
MOC: maintenance of certification
SDL: self-directed learning
